# A pandemic guided by the SARS-CoV-2 PCR test: What should the clinician know?

**DOI:** 10.4102/safp.v64i1.5492

**Published:** 2022-09-15

**Authors:** Avania Bangalee, Kreshalen Govender, Varsha Bangalee

**Affiliations:** 1 Department of Medical Virology, Faculty of Health Sciences, Prinshof Campus, University of Pretoria, South Africa; 2 National Health Laboratory Services, Johannesburg, South Africa; 3 Discipline of Pharmaceutical Sciences, Faculty of Health Sciences, University of KwaZulu-Natal, Durban, South Africa

**Keywords:** SARS-CoV-2 PCR, pandemic, COVID-19 testing, molecular diagnostic test, laboratory, COVID-19 response

## Abstract

Amidst an ever-evolving pandemic, the demand for timely and accurate diagnosis of coronavirus disease 2019 (COVID-19) continues to increase. Critically, managing and containing the spread of the disease requires expedient testing of infected individuals. Presently, the gold standard for the diagnosis of severe acute respiratory syndrome coronavirus 2 (SARS-CoV-2) infection remains the polymerase chain reaction (PCR) test. Potential vulnerabilities of this testing methodology can range from preanalytical variables to laboratory-related analytical factors and, ultimately, to the interpretation of results.

## Introduction

The coronavirus disease 2019 (COVID-19) pandemic represents a rapidly evolving public health emergency. The emergence of viral variants, a lack of diagnostic equipment and expertise coupled with inequitable vaccine access have resulted in global outbreaks. Consequently, the demand for testing continues to increase. The gold standard for the diagnosis of severe acute respiratory syndrome coronavirus 2 (SARS-CoV-2) infection remains the polymerase chain reaction (PCR) test. This test, however, requires skilled staff, laboratory infrastructure and specialised equipment. Moreover, there are numerous factors that influence the outcome and interpretation of a SARS-CoV-2 PCR test. This article will highlight potential pitfalls to consider when using this testing methodology.

## Pre-analytical factors to consider

The molecular diagnosis of COVID-19 rests on the detection of viral ribonucleic acid (RNA) in a clinical specimen. Infection begins in the upper respiratory tract (URT) and may progress to the lower respiratory tract (LRT) in more severe cases.^1^ The most common method used for respiratory specimen collection is the nasopharyngeal swab (NPS). Another specimen that may be collected is the oropharyngeal swab; however, data suggests that it is slightly less sensitive than the NPS.^2,3,4^ Saliva has been evaluated in various studies globally as it is a simple and non-invasive sample type that may be taken by a patient at home, thereby limiting contact with healthcare workers and the need for costly personal protective equipment. Moreover, saliva does not require expensive collection supplies and appears stable under variable conditions for a prolonged period.^5^ A recent systematic review of the diagnostic performance of different sampling approaches for SARS-CoV-2 reverse transcriptase polymerase chain reaction (RT-PCR) testing found that combined nasal and throat swabs gave the highest sensitivity of 97%. Lower sensitivities were obtained from saliva specimens (85%) and nasal swabs (86%) with the lowest sensitivity achieved from throat swabs (68%).^6^

The Centre for Disease Control (CDC) and the South African National Department of Health have also recently approved mid-turbinate and nasal swabs for testing.^7,8^ However, both these sample types are not routinely used in South Africa (SA) for PCR testing.

Suboptimal sample collection influences the amount of RNA detected. Specimen collection devices should have aluminium or plastic shafts as wooden shafts may contain substances that inactivate some viruses and inhibit PCR testing. The World Health Organization (WHO) recommends synthetic-tipped swabs such as rayon or nylon for SARS-CoV-2 nucleic acid detection^9^; however, shortage of collection supplies has led to the use of cotton swabs, especially in poorly resourced healthcare sectors. Whilst a recent study found that cotton does not inhibit the detection of SARS-CoV-2 RNA, further studies are required to confidently confirm this.^10^

Operator technique is another critical factor that has often contributed to false-negative results. Improper technique results in swabs not reaching the target site; the aim is to brush infected cells and secretions off the nasopharyngeal mucous membranes. This ensures that sufficient RNA is obtained from the site of initial viral replication. There has been an influx in the number of drive-through sites at which tests are being performed to improve convenience and increase the number of tests done whilst adhering to social distancing recommendations. Despite the advantages offered by a drive-through model, literature interrogating the accuracy on SARS-CoV-2 PCR results is scant, as patient and operator positioning may not be optimised for proper NPS technique.^11^

Sample type may also affect a PCR result. Patients with evidence of LRT disease may require an LRT sample, especially if the patient is tested later in the course of illness.^12^ Lower respiratory tract specimens include expectorated sputum, bronchoalveolar lavage (BAL) or endotracheal aspirates and should be submitted in clean universal containers without transport medium. These samples are especially useful when the pretest probability of disease is high and a URT specimen yielded a negative PCR result. A study evaluating the detection of SARS-CoV-2 RNA in a range of sample types found that BAL specimens showed the highest positivity rates (93%) followed by sputum (72%).^13^

Transport and storage of samples may also affect the integrity of viral RNA.^14^ Ideally, samples should reach the laboratory as soon as possible and arrive in viral or universal transport medium at 2 °C – 8 °C. Dry swabs in a sterile tube may also be submitted. Dry swabs can be sent at ambient temperature but should reach the laboratory within 48 h. Unfortunately, testing delays and backlogs have led to widespread fluctuations in transport and storage conditions. A United States-based study examining the effect of extended storage at ambient temperature on NPS found minimal and clinically insignificant impact on PCR results.^15^ Research is ongoing for molecular testing in lower-income settings under various storage conditions. In the interim, it is crucial to abide by the recommended storage and transport conditions to maintain sample integrity.

Timing of testing in relation to disease course is critical when submitting a sample to the laboratory. Viral load in the nasopharynx is highest in the immediate presymptomatic and symptomatic phase of the illness and decreases from week 3 to eventually become undetectable.^15^ Therefore, an NPS taken too early or too late in relation to clinical symptoms may cause a false negative result. Other pre-analytical errors such as sample swaps, mislabelling and contamination may also contribute to false PCR results.

## In the laboratory: Analytical factors affecting a polymerase-chain reaction test

A SARS-CoV-2 PCR test is a very sensitive molecular method of reverse transcribing SARS-CoV-2 RNA into DNA, followed by amplification of the target genomic sequence. Fluorogenic probes are used to search for the target DNA sequence which generates a fluorescent signal that increases proportionally to the amount of viral RNA present in the sample. A cycle threshold (Ct) value is obtained when the fluorescence reaches a specific threshold within a certain number of PCR cycles. A Ct value less than 40 for one or more viral gene segments is generally reported as PCR positive. There is generally an inverse correlation between Ct values and viral loads, such that lower Ct values represent higher viral RNA loads.^16^

Specific SARS-CoV-2 gene segments are targeted by different assays. Generally, most PCR assays target two or three of the envelope (*env*), nucleocapsid (*N*), spike (*S*), RNA-dependent RNA polymerase (*RdRp*) or *ORF1* genes.^17^ For any sample, these targets should take roughly the same number of PCR cycles to be detected. The Foundation for Innovative New diagnostics (FIND) is compiling a database of SARS-CoV-2 molecular assays that are commercially available or in development for the diagnosis of COVID-19, accessible at https://www.finddx.org/covid-19/pipeline/?section=molecular-assays#diag_tab. Currently, within the National Health Laboratory Service in SA, the six most widely used testing kits are presented in Table 1.

**TABLE 1:** SARS-CoV-2 molecular tests in South African public sector laboratories.

SARS-CoV-2 test	Company	Genes targeted
Alinity m	Abbott Laboratories, Abbott Park, IL, US	N, RdRp
Abbott RealTi*m*e	Abbott Laboratories, Abbott Park, IL, US	N, RdRp
Xpert^®^ Xpress	Cepheid, Sunnyvale, CA, US	E, N
cobas^®^	Roche Molecular Systems Inc., Branchburg, NJ, US	ORF1, E
TaqPath™	Thermofisher Scientific, Waltham, MA, US	S, E, N
Allplex™	Seegene Inc., Seoul, South Korea	E, N, RdRp

N, nucleocapsid; RdRp, RNA-dependent RNA polymerase; E, envelope; ORF1, open reading frame 1; S, spike; SARS-CoV-2, severe acute respiratory syndrome coronavirus 2; US, United States.

The PCR results are usually available within 24 h to 48 h of receipt and depend on patient priority (ill, hospitalised patients and healthcare workers are prioritised), the distance between the sample collection site and the testing laboratory, the volume of tests received at the laboratory and the assay(s) used.

Contamination has been a major thorn in the side of molecular assays and may be derived from two sources: cross-contamination between specimens or synthetically derived target nucleic acid.^18^ Cross-contamination from a positive sample to a negative one may occur during sample preparation for PCR. This risk increases substantially during peaks of infection when viral loads in samples are very high. During the PCR test, billions of copies of nucleic acid are generated, and these can contaminate instruments, reagents and samples. A lesser source of contamination is assay-derived, which can cause false positives and reduce the specificity of the diagnostic assay.^19^ Whilst quality assurance steps such as the use of extraction controls and negative template controls may help to detect contamination, low-level contamination may go unnoticed. This scenario can occur during a peak of infection when there are many samples containing millions of copies of viral RNA, coupled with an urgency in the laboratory to ensure that samples are processed rapidly.

The SARS-CoV-2 has a high rate of error-prone replication and penchant for recombination.^20^ As the pandemic has progressed, several genetically unique variants of concern, monitored by the WHO, have emerged globally, namely, B.1.1.7 (Alpha), B.1.351 (Beta), B.1.617.2 (Delta) and P.1 (Gamma).^21^ Because molecular tests detect a specific viral nucleic acid sequence, a mutation may affect the performance of a PCR test if it occurs in a region of the genome targeted by the test. The impact of these variants on test performance is influenced by the design of the test, the sequence of the variant and the prevalence of the variant in the population. For example, the alpha variant carries a double deletion at positions 69 and 70 on the spike protein gene (S-gene). This appears to impact detection using an assay that targets the S-gene, such as the TaqPath^TM^ COVID-19 Combo Kit.^22^ This S-gene target failure was also observed with the recent Omicron variant which contained the same mutation. Fortunately, even if a mutation impacts one of the PCR targets, most commercial PCR-based tests have two or more targets to detect SARS-CoV-2. In the case of a mutation, one may consider retesting using a test targeting different genes if COVID-19 is still suspected after receiving a negative PCR test result.

## The postanalytical phase: Interpretation of a polymerase chain reaction result

If the PCR assay detects all viral gene targets (usually two or three), then viral RNA is detected and the test is positive; if no targets are detected, then viral RNA is absent from the sample and it is reported as a negative result; if only some of the targets are present, then the laboratory takes a decision based on an agreed Ct cut-off value whether to report this result as positive or as inconclusive. An inconclusive result can be the most problematic to troubleshoot. Polymerase chain reaction-based assays can yield a weak signal or nonspecific result near the limit of detection of the assay. This high Ct value generally indicates very little viral RNA in the sample.^23^ This may be a result of a sample taken very early in the infection, intermittent viral shedding – usually at the tail-end of infection – or contamination.

It is important to remember that PCR is an extremely sensitive method and under optimal conditions can detect fewer than 10 copies of viral RNA in a clinical sample; however, it cannot distinguish between viable virus and noninfectious RNA. Virus viability appears to be brief with viral culture negative in cases with a Ct higher than 33.^24,25,26^ If one correlates Ct values with clinical progression, Ct values are lowest in the immediate presymptomatic and first week of the symptomatic phases of COVID-19.^27^ Therefore, Ct values correlate closely with a clinical history and examination. Unfortunately, these details are often omitted on a request form, so that interpretation of a result is less case-specific. Moreover, PCR can often detect viral RNA for weeks after the resolution of symptoms.^28^

Issuing binary results may create confusion by equating a sample with a very high viral load to one with a significantly lower viral load.^29^ Whilst Ct values from patient samples have demonstrated clinical utility in certain scenarios,^30^ including Ct values routinely on a PCR result report could be misleading to the clinician.^31^ It is thus critical for the requesting healthcare worker to discuss the PCR result with the clinical virologist whilst considering a patient’s history, contacts, symptoms, radiological features and previous laboratory results. From a laboratory perspective, thorough knowledge of the performance characteristics of individual PCR assays in use is necessary to accurately interpret results.^32^ Furthermore, a sample tested on different assays can yield different Ct values reflecting differences in the targets detected and the chemistry of the test used. Consequently, when considering trends in Ct values it is preferable to test samples with the same assay or platform each time.

## Conclusion

The COVID-19 pandemic has prompted a rapid upscaling of diagnostic platforms in both private and public laboratories. Whilst the PCR test currently remains the gold standard for the diagnosis of COVID-19, it is not without challenges. Pre-analytical, analytical and postanalytical factors may influence the outcome of a result. Alternative diagnostic algorithms incorporating immunoassays and novel molecular methods are currently under investigation.
